# Malformation of the Lower Extremities

**Published:** 1858-04

**Authors:** D. H. Adam

**Affiliations:** No. 119 South Wells Street; Chicago


ARTICLE VI.

### MALFORMATION OF THE LOWER EXTREMITIES.

*To the Editor of the Chicago Medical Journal.*

I have one remarkable case of anomalous structure to report, which, I hope, may not prove uninteresting to your readers.

On the 13th of December last, a woman, after an easy and natural labor, gave birth to a healthy female child. Through the care and attention of the parents, the infant has grown rapidly, is well developed, and, had not nature, by some freak, rendered it a cripple, would indeed be a fine perfect child.

The lower extremities of this child are each, as regards the knee joint, like the hind legs of quadrupeds, or rather like the arms of man. The patella are both wanting, and in their place is something like the poplitœal space, in place of which latter is a kind of elbow, so that with this joint the child can make with its legs the same motions towards its head as with its arms.

But all motions of the feet backward, as in walking, are of course impossible. The hips, and all the joints below the knee, are perfect. The growth of these limbs is symmetrical with that of the rest of the body; their sensibility is perfect, and their motions very active, which would lead one to suppose that their nervous and vascular systems are in a healthy condition.

It must be a matter of curiosity to the anatomist to know the course, origin and insertion of the muscles of these limbs, for nature must have placed them in peculiar relation with each other as well as with the arteries, which cannot be traced in this portion of the limbs.

The parents of this child, and also their two other children, are perfectly healthy. The mother, during her pregnancy, noticed nothing in her state of health, which could account for the singular malformation of her offspring. Neither can she account for it by fright, or sight of any child deformed like her own.

We have here evidently a case of deformity which is neither to be removed by the knife nor by orthopœdic apparatus. I have, however, ordered both limbs to be enveloped quite firmly with bandage, in order to prevent, as much as possible, all motion of the legs forward, and it appears to me that I have in part at least attained my object; but the further results of my treatment, I will report hereafter.

D. H. ADAM,

*Chicago, Jan.* 11, 1858. No. 119 South Wells Street.

